# Staged hybrid approach for acute type A aortic dissection: zone 2 arch replacement and completion thoracic endovascular aortic repair upon indication

**DOI:** 10.1093/ejcts/ezaf081

**Published:** 2025-03-12

**Authors:** Nesar A Hasami, Guillaume S C Geuzebroek, Foeke J H Nauta, Wilson W L Li, Michel W A Verkroost, Nabil Saouti, Robin H Heijmen

**Affiliations:** Department of Cardiothoracic Surgery, Radboud University Medical Center, Nijmegen, The Netherlands; Department of Cardiothoracic Surgery, Radboud University Medical Center, Nijmegen, The Netherlands; Department of Cardiothoracic Surgery, Radboud University Medical Center, Nijmegen, The Netherlands; Department of Cardiothoracic Surgery, Radboud University Medical Center, Nijmegen, The Netherlands; Department of Cardiothoracic Surgery, Radboud University Medical Center, Nijmegen, The Netherlands; Department of Cardiothoracic Surgery, Radboud University Medical Center, Nijmegen, The Netherlands; Department of Cardiothoracic Surgery, Radboud University Medical Center, Nijmegen, The Netherlands

**Keywords:** Type A aortic dissection, Zone 2 arch replacement, Thoracic endovascular aortic repair, Hybrid approach, Post-dissection aneurysm, Pre-emptive, Aortic remodelling, Aortic arch surgery, Landing zone, Type A dissection

## Abstract

**OBJECTIVES:**

This study evaluates a staged selective hybrid approach for acute type A aortic dissection. The approach involves a zone 2 aortic arch replacement with debranching of the brachiocephalic trunk and left common carotid artery to create a landing zone for thoracic endovascular aortic repair. This repair is performed either pre-emptively in the subacute phase to promote remodelling or electively in the chronic phase to manage aneurysm formation.

**METHODS:**

Between January 2022 and December 2023, data from patients undergoing this approach were prospectively collected and retrospectively analyzed. The study included all patients treated with zone 2 arch replacement and debranching for acute type A aortic dissection. Preoperative characteristics, surgical outcomes and follow-up interventions, including thoracic endovascular aortic repair, were assessed.

**RESULTS:**

Of the 91 patients treated for acute type A aortic dissection, 25 underwent zone 2 arch replacement. No perioperative stroke or mortality occurred in this group (mean age 62.7 years, 52% male). Eleven patients (44%) underwent thoracic endovascular aortic repair during follow-up (median interval 152 days, range 38–574). Seven repairs were performed within 6 months of the initial operation. All procedures were technically successful without complications. Early imaging showed stable or reduced aortic diameters in all thoracic endovascular aortic repair patients. In the 14 patients managed conservatively, no relevant aortic growth was observed.

**CONCLUSIONS:**

Zone 2 aortic arch replacement with debranching in acute type A aortic dissection can be performed safely. Selective pre-emptive thoracic endovascular aortic repair promoted favourable remodelling, potentially reducing the need for complex, open surgical reinterventions.

## GRAPHICAL ABSTRACT

**Figure:**
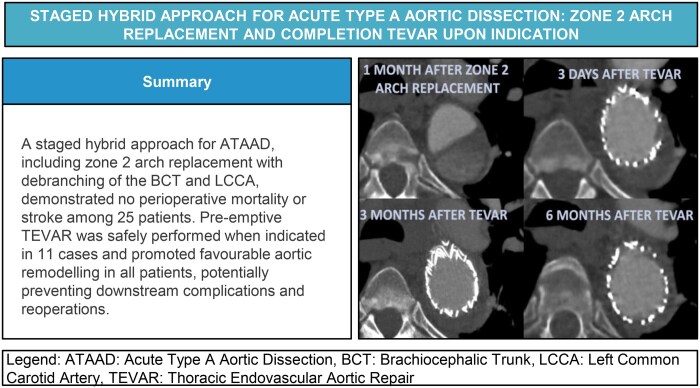

## INTRODUCTION

Acute type A aortic dissection (ATAAD) is a life-threatening condition that requires urgent surgical intervention to prevent death. Traditional surgical techniques focus primarily on repairing the ascending aorta and hemiarch, aiming to resect the primary intimal tear and restore the layers of the aortic wall. In almost 90% of cases, however, the dissection persists distal to the repaired aorta [1], which leads up to 20% post-dissection aortic dilation downstream [2]. This increases the risk of late complications such as aortic rupture and death. Post-dissection aneurysms often necessitate complex, staged open redo-surgery of the aortic arch and subsequently downstream, which carries considerable morbidity and mortality [3, 4].

To address this issue, we have progressively implemented a standardized hybrid approach in patients with ATAAD. This involves a zone 2 arch replacement (instead of hemiarch) with the distal suture line just distal to the left common carotid artery (LCCA) (zone 2), combined with debranching and proximalization of the brachiocephalic trunk (BCT) and LCCA. As a result, a 2–3 cm landing zone (LZ) is created for a potential thoracic endovascular aortic repair (TEVAR). Not as a routine, but only when deemed indicated a so-called completion TEVAR is then carried out along the entire descending thoracic aorta (DTA) to exclude the false lumen from flow [5, 6]. Aside the indication for TEVAR in case of aneurysm formation during (late) follow-up, we favour pre-emptive TEVAR within the subacute phase of the residual dissection when ‘high-risk predictors’ are identified, such as a total aortic diameter of more than 4 cm [7]. The aim of this study was to demonstrate the early results of this staged hybrid approach for ATAAD.

## METHODS

Starting in 2022, we progressively implemented zone 2 aortic arch replacement combined with proximalization of the BCT and LCCA as an alternative treatment option for patients with ATAAD. In this retrospective cohort study, we analyzed all patients who underwent this procedure between January 2022 and December 2023, using data collected prospectively in our institutional database. Patient consent was waived by the local medical ethical committee.

### Patient selection

All patients with ATAAD, who could be at risk of developing significant post-dissection dilatation later in life after the initial repair, were considered for zone 2 aortic arch replacement. However, certain factors could lead to deviation from this approach:

- DeBakey type II dissection: In DeBakey type II dissections, there is no risk of post-dissection dilation of the downstream aorta, making a less extensive procedure sufficient in most cases.
- Reoperation: A reoperation significantly increases the complexity and duration of the procedure. To avoid intraoperative complications, we were more reserved in applying this approach in patients requiring redo surgery.
- Malperfusion syndromes: In patients presenting with malperfusion of organs or limbs that persist after initiating cardiopulmonary bypass, a major issue remains even after ATAAD repair. In such cases, our priority was to keep the aortic dissection repair as short as possible, allowing for subsequent targeted intervention to address the malperfusion.
- Life expectancy of less than 5 years: If a patient’s life expectancy was too short to develop clinically relevant post-dissection dilation, there was no justification for a more extensive procedure. Given the emergency nature of ATAAD, thorough preoperative evaluation of life expectancy was often difficult and remained an estimation. In cases where significant comorbidities, such as malignancies, were known to impact life expectancy, consultation with the relevant medical specialist was always sought to obtain an estimated prognosis. Their opinion on whether to proceed with surgery and the extent of the operation was considered in the decision-making process.

The final decision on whether to operate and which surgical approach to take was made by the attending surgeon. In addition to the aforementioned factors, this decision also considered the surgeon’s experience with aortic surgery and their confidence in performing a zone 2 arch replacement.

### Operative technique

**Figure 1:**
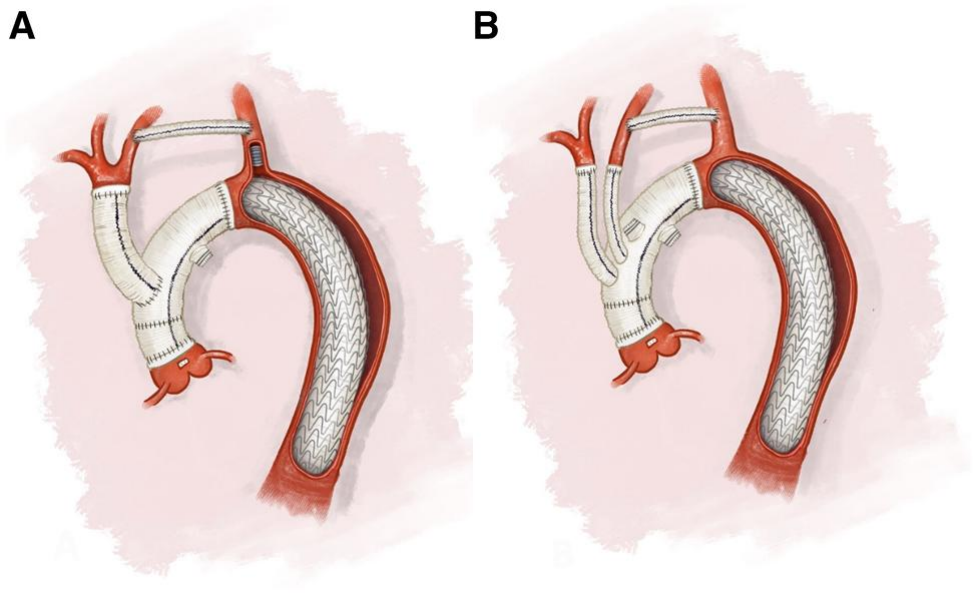
Illustration of the aorta following the hybrid surgical approach for acute type A aortic dissection, showing debranching of the brachiocephalic trunk and left common carotid artery either as an island (**A**) or as branches (**B**), along with a bypass from the left common carotid artery to the left subclavian artery and thoracic endovascular aortic repair.

Our surgical approach for ATAAD is standardized, apart from the site of arterial cannulation which depends on the clinical state and expected risk of malperfusion. While the cooling process is ongoing, the proximal part of the aorta is addressed. In cases where a supracoronary ascending replacement (SCAR) is sufficient, the intimal tear is resected, followed by resuspension of the aortic valve, and repair of the aortic layers using the neo-media technique with felt between the layers and at the outside. When deemed necessary, the aortic valve and/or root may be resected additionally. At a core temperature of 25°C, the aortic arch is opened and bilateral selective antegrade cerebral perfusion (ASCP) is installed and the left subclavian artery (LSA) occluded. The aortic arch is fully transected just distal to the LCCA (ie zone 2), and similarly reconstructed and anastomosed circular, using either a Vascutek Gelweave AnteFlo™ or Plexus™ prosthesis (Terumo Aortic, Inchinnan, UK). Antegrade flow to the lower body is resumed through the side branch of the prosthesis. Next, the prosthesis-to-prosthesis anastomosis is performed that allows removal of the cross-clamp after deairing. The procedure concludes with the debranching of the BCT and LCCA using either an ‘island’ technique with the Anteflo (Fig. 1A) separate 16 mm Dacron prosthesis or ‘separate branches’ using a Plexus (Fig. 1B) configuration (both Terumo Aortic, Inchinnan, UK). This results in a 2 to 3 cm proximal LZ for potential future TEVAR. After termination of cardiopulmonary bypass, coagulation management was guided by rotational thromboelastometry.

### Data collection

Preoperative data collected included factors such as demographics, comorbidities, laboratory values, EuroSCORE II [8], emergent ultrasound findings, symptoms and the patient’s haemodynamic and neurological status. Intraoperative details included aspects such as cannulation sites, bypass and clamp times, circulatory arrest time and procedure specifics. Postoperative outcomes included data such as complications, intensive care unit (ICU) and hospital stay duration and operative mortality (death during hospital admission or within 30 days) after ATAAD repair.

### TEVAR indication

All patients who underwent ATAAD repair are closely monitored in our outpatient clinic. A follow-up computed tomography (CT) scan of the aorta is performed within 3 months postoperatively. If early post-dissection dilatation (>5 mm) is identified and/or when the aortic diameter reaches 4 cm or greater, the patient is considered for a pre-emptive TEVAR [7]. As time progresses after the initial ATAAD repair, remodelling becomes more difficult to influence, making later pre-emptive TEVAR technically more challenging. Therefore, beyond the subacute phase (6–12 months), we carefully assess the feasibility of pre-emptive TEVAR and also consider the option of waiting until aneurysmal diameters (>5.5 cm) are actually reached.

### TEVAR procedure

In the early postoperative period, TEVAR graft sizing was based on the total aortic diameter (including both the true and false lumen) as measured on preoperative imaging. A proximal minimal oversizing of 5–10% was applied to account for potential stretching of the surgical Dacron graft. Distally, no oversizing was applied. For later postoperative interventions, sizing was highly patient-specific and depended on the time elapsed since surgery as well as the ratio between the true and false lumen. In these cases, TEVAR sizing was often more focused on the true lumen diameter, as the false lumen becomes less compliant over time.

TEVAR procedures are performed percutaneously using general anaesthesia. Prior to TEVAR placement, a central LCCA-LSA bypass is usually performed to maintain perfusion to the left arm and reduce the risk of limb or spinal cord injury (SCI). We are increasingly moving towards endovascular solutions and have also started utilizing the Thoracic Branch Endoprosthesis (TBE, Gore Medical, Flagstaff, AZ, USA). Given that the LSA is already revascularized and TEVAR is performed as a staged procedure, cerebrospinal fluid drainage (CFD) is not utilized routinely. During the TEVAR procedure, the stent graft is deployed proximally just distal to the LCCA and extends distally, typically landing just proximal to the coeliac trunk. To prevent retrograde false lumen flow, the distal stent graft is balloon-dilated (Reliant™, Medtronic Inc.) two stent rings from its distal end, the so-called Knickerbocker technique [5, 6], which creates a controlled focal expansion to seal the false lumen without excessive oversizing. Blood pressure is managed intraoperatively by temporary occlusion of the inferior caval inflow with a separate Reliant balloon [9].

### Follow-up and aortic remodelling

Follow-up data were collected via postoperative CT imaging. These scans, assessed by dedicated radiologists and aortic surgeons, were used to evaluate the state of the residual dissection downstream, including the extent of false lumen thrombosis and signs of positive remodelling. Positive remodelling was defined as a reduction of the false lumen and/or an increase in the true lumen without a corresponding increase in the total aortic diameter.

Details of follow-up procedures, imaging results and patient outcomes were systematically documented.

### Statistical analysis

All data were analyzed using IBM SPSS Statistics software [10]. Continuous variables were described using medians with range or mean with standard deviations. Categorical variables were presented as frequencies and percentages. No statistical tests for significance were performed. Descriptive statistics were used to summarize preoperative characteristics, intraoperative details and postoperative outcomes, including the frequency of complications and follow-up results. Missing data were addressed through complete case analysis.

## RESULTS

A total of 91 patients underwent surgery for ATAAD at our centre between January 2022 and December 2023. Of these, 25 patients (28%) underwent aortic arch replacement with a distal suture line in zone 2 and debranching of the BCT and LCCA, and were included in the study. The mean age was 62.7 [10] years. Further details on the preoperative characteristics are described in Table 1.

**Table 1:** Preoperative characteristics (*n* = 25)

Mean age in years	63 (10.0)
Male	13 (52%)
Mean body mass index	25.1 (3.6)
Diabetes mellitus in medical history	0 (0%)
Atrial fibrillation in medical history	1 (4%)
Myocardial infarction in medical history	1 (4%)
Pacemaker in medical history	1 (4%)
Cerebral vascular accident in medical history	1 (4%)
Transient ischaemic attack in medical history	2 (8%)
Chronic obstructive pulmonary disease in medical history	0 (0%)
Mean creatinine at presentation^a^	103 (31)
Mean eGFR at presentation^a^	63 (18)
Mean EuroScore II	6.8 (3.1)
Pericardial effusion present on echocardiogram^a^	5 (20%)
Aortic valve regurgitation present on echocardiogram^a^	None 10 (20%) Mild 3 (12%) Moderate 7 (28%) Severe 4 (16%)
Left ventricular function on echocardiogram	>50% 24 (96%) 31–50% 1 (4%)
Malperfusion^b^	5 (20%)
Haemodynamics	Stable 20 (80%) Hypotensive 2 (8%) Shock due to tamponade 3(12%)
Neurology	Intact 20 (80%) Impaired without focal deficit 2 (8%) Impaired with focal deficit 3 (12%)

Values are presented as mean (standard deviation) or as absolute numbers (percentages).

a Data missing: *n* = 1.

b One patient had malperfusion of the right leg, right kidney and threatened visceral perfusion; 1 patient had right arm malperfusion; 1 patient had left arm malperfusion, 1 patient had left leg malperfusion and 1 patient had myelum malperfusion causing paraparesis.

In 3 patients (12%), the dissection was limited to the aortic arch. In these cases, zone 2 aortic arch replacement was performed to completely resect the dissected segment. The majority of patients, 14 out of 25 (56%), underwent a SCAR, and all patients in this group retained their native aortic valve. The remaining 11 patients (44%) underwent a Bentall procedure, of which 7 patients (28%) received a biological composite graft and four patients (16%) a mechanical aortic valve prosthesis. Debranching of the BCT and LCCA was performed using the island technique (Fig. 1A) in 19 patients (76%), while the other 6 patients (24%) received separate branches (Fig. 1B). Further details on the intraoperative variables are described in Table 2.

**Table 2:** Intraoperative variables (*n* = 25)

Arterial cannulation	Femoral artery 15 (60%) Ascending aorta 4 (16%) Right axillary artery 6 (24%)
Venous cannulation	Femoral vein 3 (12%) Right atrium 22 (88%)
Mean cardiopulmonary bypass time	296 (98) min
Mean aortic cross-clamp time	169 (57) min
Mean antegrade cerebral perfusion time	135 (49) min
Mean circulation arrest time	42 (13) min
Supracoronary ascending aorta replacement	14 (56%)
Aortic valve replacement	None 14 (56%) Biological prosthesis 7 (28%) Mechanical prosthesis 4 (16%)
Bentall	11 (44%)
Technique used for the proximalization of the brachiocephalic trunk and the left common carotid artery	Island technique 19 (76%) Separate branches 6 (24%)
Type of prosthesis used for distal anastomosis	AnteFlo 19 (76%) Plexus 6 (24%)
Concomitant coronary artery bypass grafting^a^	5 (20%)
Second run of cardiopulmonary bypass^b^	2 (8%)

Values are presented as mean (standard deviation) or as absolute numbers (percentages).

a Reasons for concomitant coronary artery bypass grafting (CABG): 3 patients had a severely dissected ostium of the right coronary artery; 1 patient had unexplained right-sided heart failure after cardiopulmonary bypass (CPB); and 1 patient showed persistent inferior wall dyskinesia during reperfusion.

b The second CPB run in both cases was due to unexplained right-sided heart failure, prompting pragmatic CABG.

During the initial hospital stay, 8 patients (32%) required re-thoracotomy for various reasons. Four of these patients (16%) were reoperated due to excessive postoperative drain leakage within the first 24 h in the ICU. Two patients (8%) because of pericardial effusion which was detected during a routine transthoracic echocardiogram control on the ward. In 1 patient, the sternum was intentionally left open, with surgical gauze placed during the primary operation due to inadequate coagulation. This patient’s sternum was successfully closed after a few days. In another patient, initial severe delirium was suspected to be caused by global brain hypoperfusion due to a severely stenosed dissected true lumen of the BCT and an LCCA that was torn after multiple attempts to revascularize the vessel in a Marfan’s patient. In this case, a reoperation was performed at day 4 postoperatively, which consisted of an interposition graft from the Plexus graft to the LCCA bifurcation. The neurological outcome was uneventful. All patients underwent routine postoperative echocardiography on the ward. Pericardial effusion was observed in 11 cases (44%), leading to intervention in 10 cases (40%) (2 resternotomies [8%]—the same 2 patients previously mentioned—4 subxiphoid drainages [16%], and 4 percutaneous drainages [16%]). Haemodynamic compromise due to pericardial effusion was observed in 5 of these patients (20%).

No new postoperative stroke occurred. One patient who presented preoperatively with paraparesis (receiving urgent CFD post ATAAD repair) showed improvement at the time of discharge and was able to walk with crutches. Three patients required temporary postoperative dialysis, but none of them required dialysis at the time of discharge. For a detailed overview of the postoperative outcomes during the initial admission, see Table 3.

**Table 3:** Postoperative outcomes during initial hospital stay (*n* = 25)

Low cardiac output syndrome	0 (0%)
Stroke (transient and non-transient)	0 (0%)
Spinal cord injury	0 (0%)
Delirium	16 (64%)
Mean highest creatinine^a^	155 (83)
Dialysis	Temporary 3 (12%) Permanent 0 (0%)
Mean duration of intensive care unit stay in days^a^	8 (6)
Mean duration of hospitalization in days	18 (9)
Operative mortality	0 (0.0%)

Values are presented as mean (standard deviation) or as absolute numbers (percentages).

a Data missing: *n* = 1.

All patients were followed up with CT scans, with the first postoperative scan performed during the initial hospital stay and the second scan conducted on an outpatient basis. Based on CT-scan findings, 11 patients (44%) underwent a secondary TEVAR during the study interval: all were placed pre-emptively (in the presence of so-called ‘high-risk predictors’ for late dilatation [7]). Of the 11 TEVAR procedures, the majority (7/11, 64%) were performed within 6 months after type A repair, 1 procedure (9%) was performed between 6 months and 1 year, and the remaining 2 procedures (18%) were performed more than 1 year after the initial operation. The median interval for TEVAR during follow-up was 152 days (range 38–574).

Nine of the TEVAR procedures had concomitant LCCA–LSA bypass, while 1 patient already had a trans-pleural extra-anatomic bypass of the LSA performed at the initial ATAAD operation. In 1 patient, the LSA was revascularized using a TBE as a proximal stent graft. Only the GORE^®^ Conformable Thoracic Aortic Graft (CTAG), Relay^®^ PRO Thoracic Stent-Graft System and TBE were used as stent grafts, with GORE CTAG typically placed proximally (7/11, 70%) and Relay PRO distally (9/11, 90%).

**Figure 2:**
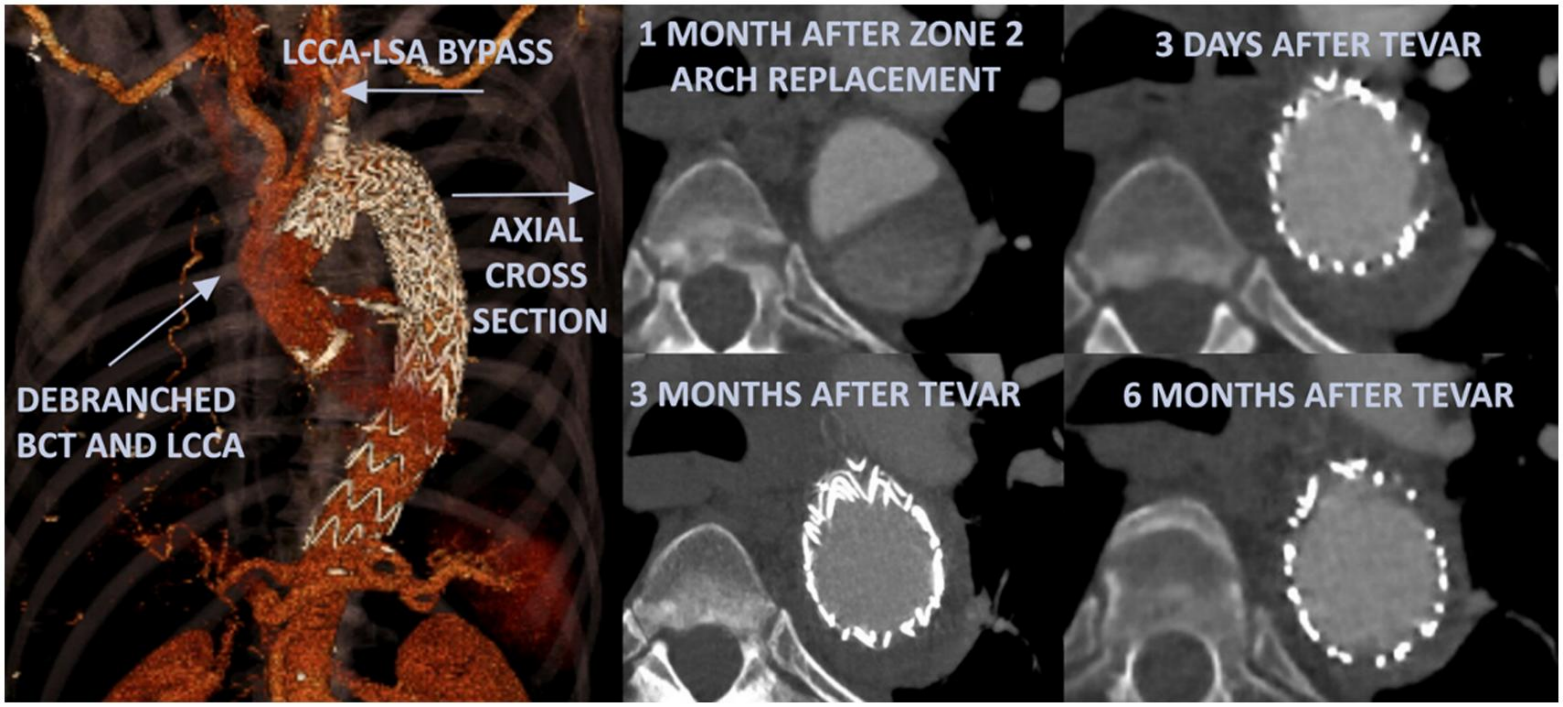
CT reconstruction with cross-sections at different time intervals in a patient after zone 2 arch replacement with debranching of the brachiocephalic trunk and left common carotid artery for acute type A aortic dissection, followed by pre-emptive thoracic endovascular aortic repair.

No patients experienced stroke or SCI, and there were no cases of mortality. Early postoperative CT scans after TEVAR showed complete antegrade exclusion of the false lumen with true lumen expansion in all these patients. Later follow-up CT scans showed no negative aortic remodelling in all patients (see Fig. 2 for exemplary CT images of a patient with positive remodelling following TEVAR), with a decreased total aortic diameter in 4 patients and a stable diameter in 7. The remaining 14 patients who underwent zone 2 arch replacement only, the diameter of the DTA remained stable during the study interval and as a result, are conservatively managed yet and kept in close follow-up.

## DISCUSSION

Our data on the initial 25 patients show that zone 2 arch replacement in ATAAD can be performed with low surgical risk in selected patients. In our current study, we found no mortality nor adverse neurological outcomes with this strategy. This approach facilitates an LZ for TEVAR that can be used pre-emptively in the subacute phase to induce remodelling and prevent late aneurysm formation, as well as electively treat late aneurysms. The presence of the LZ simplifies the management of future aortic problems, potentially avoiding complex, open redo arch and descending aorta operations This study was primarily designed to assess the feasibility and early outcomes of the zone 2 arch replacement approach. As a result, we did not systematically collect reintervention rates for the remaining 66 patients who underwent alternative surgical strategies for ATAAD. However, to further evaluate the long-term effectiveness of our approach, we plan to report separately on our full cohort of type A dissections, including a comparative analysis of this staged zone 2 strategy (with pre-emptive TEVAR upon indication) versus other techniques in terms of aortic remodelling and reinterventions.

This approach was first technically described by Glauber *et al.* [11] in 2011 and, in a different form, by Desai *et al.* [12], who utilized single-branched TEVAR as a routine follow-up procedure for ATAAD, providing an alternative to the frozen elephant trunk (FET). In our approach, completion TEVAR is applied only when indicated, as only a limited percentage of patients will develop downstream complications. The LZ enables pre-emptive TEVAR in the subacute phase to prevent late aneurysm formation by inducing remodelling. By performing TEVAR several months after the initial ATAAD operation, we can safely extend the coverage to the entire DTA in a hybrid operating room under optimal conditions with the most optimal stent grafts available in terms of length, design and safety for SCI prevention. Importantly, a considerable percentage of patients do not require additional TEVAR, which is cost-effective compared to routine FET or routine completion TEVAR. Should an aneurysm later develop in the yet conservative group, the proximal LZ enables a simpler and safer open, endovascular or hybrid intervention. Our approach, using zone 2 arch replacement with completion TEVAR upon indication, was briefly described earlier [13]. Desai *et al.* have shown that additional TEVAR following zone 2 arch replacement is safe and effective, even with the recently introduced single-branched stent graft like TBE, which negates the need for an LSA bypass. An LSA bypass, however, remains a feasible (and cost-effective) alternative offering favourable long-term results [14]. Given the full-length exclusion of the DTA false lumen in extensive completion TEVAR, we believe LSA revascularization is critical to reduce SCI risk. Since TEVAR is staged, and delayed to the subacute phase of the dissection, the risk of SCI is already lower than with immediate intervention during ATAAD repair, and consequently routine CSF drainage is not applied by us. In our experience, no SCI occurred after ATAAD repair with the zone 2 arch replacement, nor after completion TEVAR.

Comparing the zone 2 arch replacement with FET in ATAAD, we feel that, while FET pursues similar remodelling goals, it is a more complex procedure in the acutely dissected, non-dilated arch, particularly for less experienced aortic surgeons. It is also costly and may not always be necessary to prevent downstream complications (only a limited percentage of patients dilate over time). Published outcomes demonstrate high mortality rates with FET [15] and risk of SCI [16]. Although FET induces effective remodelling of the DTA at the stented site, the currently available FET devices are limited to the proximal DTA, while our approach addresses the entire DTA under optimal conditions in a hybrid OR. The hyper-acute versus subacute phase of intervention is also important; in the hyper-acute phase, the membrane is exceptionally fragile, increasing the likelihood of a distal stent-induced new entry (dSINE) [17]. Therefore, FET may be a viable option in ATAAD with severe acute distal malperfusion, but as a routine approach, it may be too complex, risky, costly and strictly not necessary. The zone 2 arch replacement offers a technically simpler procedure that can be performed by surgeons with less experience in complex aortic cases. Thus, we believe that the zone 2 arch approach with TEVAR upon indication is a viable alternative to FET.

In our study, 11 patients underwent TEVAR, all pre-emptively due to early dilation to over 4 cm (a recognized high-risk predictor for late aortic events in Type B dissection, which we also apply here for residual dissection). However, long-term risks such as mortality and aneurysm formation are significantly higher following type B dissection. The actual high-risk predictors for residual dissection after type A repair remain unclear and warrant further study to refine the indications for completion TEVAR. For now, our approach relies on insights from TEVAR in type B dissections. Notably, all 11 patients in the first follow-up showed promising remodelling downstream. Further follow-up is necessary to determine if this intervention will also prevent late aneurysm formation which is the rationale for this early intervention. It also remains unknown if stent-grafting the entire DTA, possibly due to compliance changes, could increase the risk of abdominal dilation. This is an area for further study.

In this endovascular era, endovascular arch treatment after a hemiarch procedure has been documented [18] but requires a good zone 0 LZ. TEVAR with branched arch repair remains complex, costly, and carries high stroke and reintervention risks [18]. In contrast, the zone 2 arch approach allows for simpler TEVAR, with a slight increase in surgical complexity but a standardized pre-emptive TEVAR option, unlike complex branched arch interventions. Complex branched arch interventions are not performed pre-emptively, whereas our standard TEVAR in this approach is.

Regarding device choice, we selected the cTAG proximally due to its short nose cone as longer nose cones on other devices can make it challenging to navigate deeply into the ascending aorta and fully utilize the LZ. For the distal segment, we avoid cTAG in favour of stent grafts with larger separate stents, which better facilitate the Knickerbocker technique. With cTAG (small connected stents), dSINE risk is increased upon balloon dilation.

During the study period, 28% of ATAAD cases underwent zone 2 arch replacement. This was due to the gradual introduction of the technique, initially adopted by experienced aortic surgeons and later by less experienced colleagues. As a result, its use has increased and may eventually cover up to 3 quarters of cases (excluding DeBakey II and advanced age patients).

## CONCLUSION

The hybrid approach for treating ATAAD with zone 2 arch replacement and proximal reimplantation of the BCT and LCCA has demonstrated promising results. This technique provides a stable platform for future endovascular interventions and may reduce the need for complex redo surgeries. In our cohort, postoperative outcomes were favourable, with no new neurological complications or operative mortality observed, both after the initial ATAAD repair and the secondary TEVAR. Stable or decreased aortic diameters were observed in all patients who underwent secondary TEVAR, indicating the long-term benefits of this staged approach. Our findings suggest that this strategy offers a viable and tailored alternative for managing the long-term risks associated with ATAAD, particularly for patients at risk of downstream aortic dilation and reintervention.

## Data Availability

Data underlying this article will be shared on reasonable request to the corresponding author.

## ABBREVIATIONS

ATAAD: Acute type A aortic dissection
BCT: Brachiocephalic trunk
CFD: Cerebrospinal fluid drainage
CPB: Cardiopulmonary bypass
CT: Computed tomography
CTAG: Conformable thoracic aortic graft
CSF: Cerebrospinal fluid
DTA: Descending thoracic aorta
dSINE: Distal stent-induced new entry
FET: Frozen elephant trunk
ICU: Intensive care unit
LCCA: Left common carotid artery
LSA: Left subclavian artery
LZ: Landing zone
SCAR: Supracoronary ascending replacement
SCI: Spinal cord injury
TEVAR: Thoracic endovascular aortic repair
